# A fast online questionnaire for screening mental illness symptoms during the COVID-19 pandemic

**DOI:** 10.1038/s41398-022-02086-7

**Published:** 2022-08-04

**Authors:** Fang Chen, Weizheng Yan, Vince D. Calhoun, Linzhen Yu, Lili Chen, Xiaoyi Hao, Leilei Zheng

**Affiliations:** 1 Department of Psychiatry, Second Affiliated Hospital, School of Medicine, Zhejiang University, Hangzhou, China; 2 Tri-Institutional Center for Translational Research in Neuroimaging and Data Science (TReNDS), Georgia State University, Georgia Institute of Technology, Emory University, Atlanta, USA

**Keywords:** Psychiatric disorders, Predictive markers, Human behaviour

## Abstract

The COVID-19 pandemic has caused massive effects on the situation of public mental health. A fast online questionnaire for screening and evaluating mental symptoms is urgent. In this work, we developed a new 19-item self-assessment Fast Screen Questionnaire for Mental Illness Symptoms (FSQ-MIS) to quickly identify mental illness symptoms. The FSQ-MIS was validated on a total of 3828 young adult mental disorder patients and 984 healthy controls. We applied principal component analysis (PCA), receiver operating characteristic (ROC) curve, and general log-linear analysis (GLA) to evaluate the construct and parallel validity. Results demonstrate that the proposed FSQ-MIS shows high test-retest reliability (0.852) and split-half reliability (0.844). Six factors obtained using PCA explained 54.3% of the variance and showed high correlations with other widely used scales. The ROC results (0.716–0.983) revealed high criterion validity of FSQ-MIS. GLA demonstrated the advantage of FSQ-MIS in predicting anxiety and depression prevalence in COVID-19, supporting the efficiency of FSQ-MIS as a tool for research and clinical practice.

## Introduction

In psychiatric practice, the most common mental illnesses include anxiety, depression, bipolar disorder, schizophrenia, and obsessive-compulsive disorder in Chinese population [1]. The rating scales, which are incorporated into clinical practice, offer psychiatrists important clinical information that may have been missed or not addressed in the clinical interview. The information can also be used to follow progression of symptoms and effectiveness of treatment.

However, the number of competing scales can make choosing a measure difficult [2]. Most of the self-rating scales target specific symptoms such as the Patient Health Questionnaire (PHQ-9) for depression [3], Generalized Anxiety Disorder Questionnaire 7-item scale (GAD-7) for anxiety [4], and Mood Disorder Questionnaire (MDQ) for hypo-mania [5]. These scales help quantify disease severity after a brief interview with a doctor. Symptom Checklist-90-Revised (SCL-90-R) [6] is a widely used self-rating scale which covers a relatively broad range of psychological problems and symptoms including anxiety, depression, psychosis, and obsession. However, the SCL-90-R contains 90 questions and takes around twenty minutes to complete. Besides, the SCL-90-R does not evaluate the manic or cognitive symptoms. Therefore, there is a need for developing a time-saving and comprehensive self-rating symptom screening scale. Moreover, the fast-screening questionnaire would also provide useful information for recommending of illness-specific scales following the initial clinical interview.

The COVID-19 pandemic has caused a heavy burden on mental health [7]. Home quarantine and other social distancing rules are making people feel isolated, stressed, anxious or even panicky. Anxious and depressive moods have been reported as a main health concerns in this pandemic [8, 9]. In addition, because of the quarantine rules, those who have mental problems are less likely to leave home for help. Therefore, an online fast-screening questionnaire that can access easily may help them to evaluate their current mental status and decide whether to visit a doctor.

The popularity of smartphones and cloud services makes it possible for quickly transfer, analyze, and store data. Through the internet, the doctors can receive the initial general screening report from the patients, quickly assess the main symptoms, and offer more precise interviews and diagnoses when patient comes to the clinic. In addition, a fast online screen questionnaire will also benefit large-scale epidemiological surveys.

In the present study, we propose a fast online screen questionnaire for symptom identification of mental illness symptoms (FSQ-MIS). To the best of our knowledge, there is the first online questionnaire that covers most of the major mental symptoms. We tested the reliability and validity on a large cohort of Chinese participants. The proposed FSQ-MIS was also used to predict the trend of anxiety and depressive events during the pandemic, and the parallel validity with DSM-5 diagnosis were also obtained.

## Materials and methods

### Participants and procedure

A total of 3828 young adult mental disorder patients (mean age = 25.4, SD = 4.0; male/female = 1803/2025) were recruited in the outpatient psychiatric department at Second Affiliated Hospital, Zhejiang University School of Medicine from December 2019 to November 2021. The normal controls were 984 medical staff and students (mean age = 25.0, SD = 4.7; male/female=476/507). There exists no significant age and gender difference between patients and controls (*t* = 0.356, *p* = 0.722; *x*^2^ = 0.539, *p* = 0.463). All the 4812 participants filled out the FSQ-MIS.

The patients were also evaluated using other clinical classic scales, including Hamilton Anxiety Rating Scale (HARS, *n* = 1703), Hamilton Depression Rating Scale (HDRS, *n* = 1849), Pittsburgh Sleep Quality Index (PSQI, *n* = 2363), Yale-Brown obsessive-compulsive scale (YBOCS, *n* = 544), Positive and Negative Syndrome Scale (PANSS, *n* = 145) and Bech-Rafaelsen Mania Scale (BRMS, *n* = 40).

According to DSM-5, 2940 subjects were diagnosed with depressive/anxiety-related disorder; 1814 subjects were diagnosed with sleep disturbance; 525 subjects were diagnosed with psychosis; 463 subjects were diagnosed with obsessive-compulsive disorders, and 456 subjects were diagnosed with bipolar or excitation status.

Each patient received a face-to-face interview with the examiner and completed the questionnaires online using their smartphones. FSQ-MIS and PSQI are self-rating scales, which were fulfilled by the participants using their own smartphones. HARS, HDRS, PANSS, YBOCS, and BMRS were examiner-rating scales, which were completed by the professional senior psychological assessors. The information was encrypted and stored in the hospital information system. The study protocol was approved by the Ethics Review Committee of the Second Affiliated Hospital of Zhejiang University and the informed consent was obtained from all subjects.

### Measurements

The FSQ-MIS is a 19-item self-rating scale for assessing symptoms within the last week. It is a force-choice questionnaire with YES/NO responses (YES = 1, NO = 0). The specific items, which reference the SCl-90 [6] and MDQ [5], are listed in Table 1.

**Table 1:** Items of FSQ-MIS and the comparison with six scales.

FSQ-MIS Item	Question	FSQ-MIS Item	Question
1	Difficulty falling asleep	**11**	Check things over and over again
2	Low mood	**12**	Feel that others can know your private thoughts
3	Decreased interest	**13**	Washing hands, counting, or touching something repeatedly
4	Being agitated	**14**	Feel restless, anxious, and preoccupied
5	Memory loss	**15**	Several times I felt flustered, chest tightness and suffocation
6	Worried and nervous	**16**	Often wake up during sleep
7	Hear voices that others can’t hear	**17**	Attention distracted
8	Someone tried to persecute me	**18**	I feel my brain is very active
9	Feel your energy declined, and activities slow down	**19**	Spent a lot of money recently
10	Headache		

Scales	Rating approach	Averaged rating Time (minute)	Reliability/validity coefficient	
			on Chinese norm	In this study
FSQ-MIS	self-rating	5	Verified in this study	
HARS	examiner-rating	15–20	0.93^10^*	0.91*
HDRS	examiner-rating	15–20	0.88–0.99^11^*	0.95*
PSQI	self-rating	20	0.84^12^*	0.88*
YBOCS	examiner-rating	30	0.75^13^*	0.83*
PANSS	examiner-rating	30–50	0.73–0.83^14^*	0.78–0.82*
BRMS	examiner-rating	20	0.92 ^15^#	0.89*

*FSQ-MIS* Fast-Screening Questionnaire for Mental Disorder, *HARS* Hamilton Anxiety Rating Scale, *Hamilton HDRS* Depression Rating Scale, *PSQI* Pittsburgh Sleep Quality Index, *YBOCS* Yale-Brown obsessive-compulsive scale, *PANSS* Positive and Negative Syndrome Scale, *BRMS* Bech-Rafaelsen Mania Scale.

*Cronbach’s alpha coefficient, ^#^criterion validity coefficient with Global Assessment Scale.

The HARS is a widely used classical anxiety symptom rating scale consisting of 20 items for rating psychotic and somatic anxiety. The HDRS is a classical depressive symptom rating scale. In present study, we used the 24-item version. The PSQI is a self-rating scale for the estimation of sleep disturbance. The YBOCS is a 10-item clinical examiner-rating scale for assessing the severity of obsessive and compulsive symptoms. The PANSS is a 30-item examiner-rating scale for assessing psychotic symptoms, including positive, negative, aggressive and general pathological symptoms. The BRMS is a scale for assessing manic/hypomanic symptoms. The above six measurements have been verified high reliability and validity both in the Chinese norm [10–15] and the present study (Table 1). The total scores of these six scales were used for verifying the criterion-related validity of FSQ-MIS.

The FSQ-MIS covers the core symptoms of anxiety, depression, bipolar disorder, psychosis, and obsessive-compulsive disorder. The comparison between FSQ-MIS and the six classical rating scales was shown in the lower half of the Table 1 [10–15].

### Data analysis

We first used the Kaiser–Meyer–Olkin (KMO) Measure of Sampling Adequacy and Bartlett’s Test of Sphericity value to determine the suitability of the data for component analyses. Principal component analysis (PCA) with varimax rotation was then conducted for analyzing the construct validity of the FSQ-MIS. The receiver operating characteristic (ROC) curve and area under curve (AUC) were calculated to evaluate the discriminant validity of the FSQ-MIS in screening mental disorders according to DSM-5 criteria. Pearson correlation coefficient was used to evaluate the criterion validity between FSQ-MIS and other scales, including HARS, HDRS, PSQI, PANSS, PSQI, and BRMS. We defined the factor which had the equal weight in anxious and depressive symptoms as the FSQ-MIS-F1. Subsequently, the general log-linear analysis was conducted to estimate the main effect of time stages in predicting of having anxiety and depression in mental disorder population of this study, which was screened by FSQ-MIS-F1 or diagnosed by DSM-5 respectively. All the analyses were conducted using SPSS 26.0 (IBM Corp, Armonk, NY, USA).

## Results

### Reliability

A subset of the patients (*n* = 134) received the FSQ-MIS retest, the one-week test-retest reliability in patients was 0.852 (*p* < 0.001). The split-half reliability (odd items vs. even items) for the 4812 participants was 0.844 (*p* < 0.001).

### Validity

#### Construct validity

The KMO value for the FSQ-MIS was 0.890 and the result of Bartlett’s test of Sphericity was above the satisfactory level (*x*^2^ = 13011.027, *p* < 0.001), indicating that the sampling was adequate for factor analysis.

The principal component analysis showed the 6-factor model explained 54.28% of the variance. The varimax rotated matrix is listed in Table 2. We define the 6 factors as anxiety/depression symptom (F1), cognitive deficit (F2), sleep disturbance (F3), psychotic symptom (F4), obsessive-compulsive symptom (F5), and excitation symptom (F6). The F1 consisted of item 2, 3, 4, 6, 14, and 15. The F2 consisted of item 5,9, and 17. The F3 consisted of item 1, 10, and 16. The F4 consisted of item 12, 7 and 8. The F5 consisted of item 11 and 13. The F6 consisted of item 18 and 19.

**Table 2:** Factor analysis matrix of FSQ-MIS.

	Components					
	F1	F2	F3	F4	F5	F6
Item 1	0.157	0.065	0.736	0.027	−0.024	0.103
Item 2	0.692	0.289	0..095	0.095	−0.041	0.012
Item 3	0.575	0.457	0.095	0.058	−0.046	−0.038
Item 4	0.627	0.129	0.095	0.054	0.059	0.156
Item 5	0.053	0.777	0.150	0.041	0.137	0.018
Item 6	0.688	−0.012	0.069	0.012	0.172	−0.004
Item 7	−0.068	0.137	0.238	0.597	0.141	−0.006
Item 8	0.153	-0.019	-0.014	0.745	0.055	0.091
Item 9	0.458	0.544	0.127	0.054	0.035	−0.021
Item 10	0.122	0.253	0.490	0.173	0.169	−0.060
Item 11	0.187	0.125	0.014	0.009	0.802	0.094
Item 12	0.191	0.071	−0.005	0.648	0.076	0.099
Item 13	0.058	0.064	0.092	0.280	0.723	0.009
Item 14	0.600	0.077	0.225	0.227	0.192	0.002
Item 15	0.416	0.073	0.362	0.292	0.064	0.034
Item 16	0.127	0.074	0.753	-0.001	0.021	0.066
Item 17	0.341	0.588	0.110	0.101	0.091	0.076
Item 18	0.073	−0.152	0.144	0.029	0.101	0.803
Item 19	0.027	0.352	−0.020	0.220	−0.007	0.599

Anxiety and depression are two mental disorders having a high comorbidity rate [16]. The F1 factor was consistent with this clinical conclusion. The factor analysis exhibited that item 2, 3, 4, 6, and 14 has high weights in the component F1 (Table 2). Moreover, the F1 had significant correlations with both HDRS and HARS (Table 3), indicating that F1 reflects both anxiety and depressive symptoms. Therefore, we define this factor as FSQ-MIS-F1 and used it in the following general log-linear analysis for prediction.

**Table 3:** Criterion validity of factors in FSQ-MIS.

	F1	F2	F3	F4	F5	F6
HDRS	0.695**	0.511**				
HARS	0.654**	0.695**				
PSQI		0.314**	0.460**			
PANSS		0.508**		0.522**		
YBOCS		0.228**			0.528**	
BRMS		0.331**				0.482**

**2-tailed significance at *p* < 0.001; significant correlation coefficients were presented. *HDRS* Hamilton depression rating scale, *HADS* Hamilton anxiety rating scale, *PSQI* Pittsburgh sleep quality index, *PANSS* Positive and negative syndrome scale, *Y-BOCS* Yale-Brown obsessive-compulsive scale, *BRMS* Bech-Rafaelsen Mania Rating Scale. F1 = anxiety and depression symptom; F2 = cognitive deficit; F3 = sleep disturbance; F4 = psychotic symptom; F5 = obsessive-compulsive symptom; F6 = excitation symptom.

#### Criterion-related validity

In this study, we used HDRS, HARS, PSQI, PANSS, YBOCS and BRMS for testing the criterion-related validity of FSQ-MIS. We calculated the correlation between the PCA-derived 6 factors and the 6 popular criterion scales. As shown in Table 3, F1 is highly correlated with both HDRS and HARS. F2 is highly correlated with all the popular criterion scales, indicating its potential in evaluating cognitive status. F3, F4, F5, and F6 were significantly correlated with PSQI, PANSS, YSOCS, and BRMS respectively. These results indicated the 5 factors (F1, 3, 4, 5, 6) had high discriminative power in identifying anxiety/depression, insomnia, psychotic, obsessive, and excitation symptoms.

#### Receiver operating characteristic analysis

**Fig. 1:**
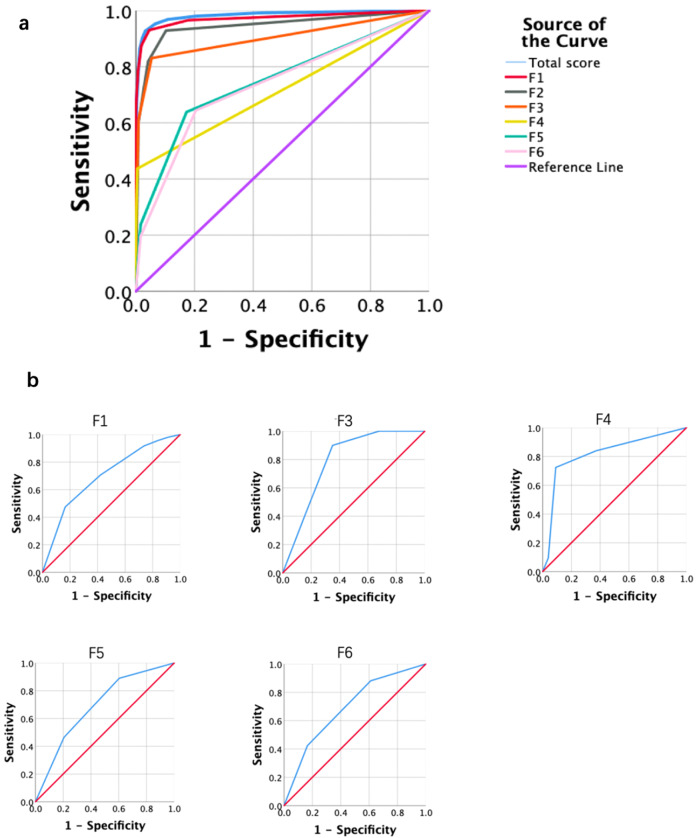
ROC analysis. **a** ROC curves of FSQ-MIS total score and factor scores for discriminate patients and healthy controls. Total score for discrimination, AUC = 0.983 ± 0.002, *p* < 0.001, 95%CI [0.980, 0.986]. F1 for discrimination, AUC = 0.972 ± 0.002, *p* < 0.001, 95%CI [0.968, 0.977]. F2 for discrimination, AUC = 0.944 ± 0.004, *p* < 0.001, 95%CI [0.937, 0.952]. F3 for discrimination, AUC = 0.902 ± 0.004, p < 0.001, 95%CI [0.893, 0.911]. F4 for discrimination, AUC = 0.716 ± 0.007, *p* < 0.001, 95%CI [0.702, 0.731]. F5 for discrimination, AUC = 0.748 ± 0.008, *p* < 0.001, 95%CI [0.733, 0.763]. F6 for discrimination, AUC = 0.734 ± 0.008, *p* < 0.001, 95%CI [0.719, 0.750]. **b** ROC curves for measuring the performance of FSQ-MIS-Factors (F1, F3, F4, F5, F6) in identifying the mental disorders. F1 predicts anxiety and depression related diagnosis, AUC = 0.700 ± 0.010, *p* < 0.001, 95%CI [0.681, 0.720]; F3 predicts sleep disturbance related diagnosis, AUC = 0.791 ± 0.007, *p* < 0.001, 95%CI [0.777, 0.806]; F4 predicts psychosis related diagnosis, AUC = 0.820 ± 0.011, *p* < 0.001, 95%CI [0.799, 0.842]; F5 predicts obsessive-compulsive related diagnosis, AUC = 0.692 ± 0.012, *p* < 0.001, 95%CI [0.667, 0.716]; F6 predicts excitation symptom related diagnosis, AUC = 0.692 ± 0.013, *p* < 0.001, 95%CI [0.667, 0.717].

To estimate the validity of FSQ-MIS in discriminating patients with mental disorders from healthy controls, ROC curve analysis was conducted for quantification. The FSQ-MIS total score and each factor score were used for discrimination. As shown in Fig. 1a, the AUC ranges from 0.716 to 0.983, indicating satisfactory discrimination validity in distinguishing patients from healthy individuals.

As shown in Fig. 1b, to further evaluate the diagnostic validity of FSQ-MIS in screening different mental illness symptoms, we plotted the ROC curves of F1, 3, 4, 5, 6 in predicting anxiety and depression-related diagnosis (e.g., anxiety, depression, anxiety/depressive state), sleep disturbance related diagnosis (e.g., insomnia, narcolepsy), psychosis related diagnosis (e.g., brief psychotic disorder, delusion disorder, schizophrenia), obsessive-compulsive related diagnosis (e.g., obsessive-compulsive disorder, body dysmorphic disorder, eating disorder) and excitation symptom-related diagnosis (e.g., cyclothymic disorder, bipolar disorder, excitation state). The AUCs ranged from 0.692 to 0.820, indicating satisfactory discrimination validity of the FSQ-MIS.

### Application in evaluating anxiety and depression situation during COVID-19

**Fig. 2:**
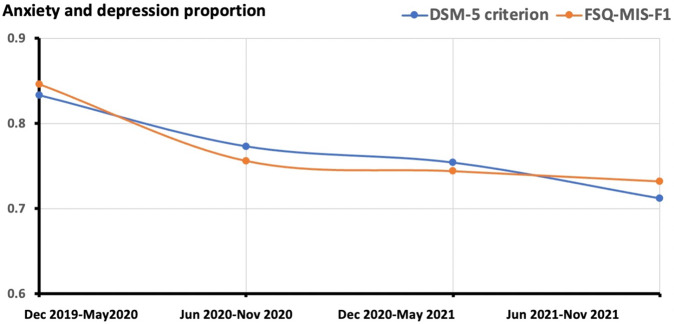
General loglinear analysis of anxiety and depression in the four COVID-19 stages. FSQ-MIS-F1 = Factor1 of the fast-screening questionnaire for mental illness symptoms. The results show that the anxiety and depression decreased with the relief of the pandemic in China mainland, and the FSQ-MIS exhibits high parallel validity with DSM-5.

Anxiety and depression were the most common symptoms during the pandemic. our proposed FSQ-MIS showed advanced performance in predicting the anxiety and depression proportion during the COVID-19 pandemic. The two years were manually divided into four stages: from December 2019 to May 2020 (stage I), from June 2020 to November 2020 (stage II), from December 2020 to May 2021 (stage III), and from June 2021 to November 2021(stage IV). Stage I was the outbreak and most serious period of the COVID-19 pandemic in China. After the period, the pandemic gradually faded away. The proportion of having anxiety and depression screened by FSQ-MIS-F1 or DSM-5 diagnosis (e.g., anxiety, depression, anxious and depressive status) were used as structure variables in a general loglinear analysis to predict the COVID-19 effects. The result revealed stage I contributed most to anxiety/depression occurrence. The subsequent stage II and stage III had less influence on anxiety/depression occurrence, and stage IV had almost no effect on the occurrence (Table 4 and Fig. 2). The results demonstrated that anxiety and depression decreased with the relief of the pandemic. In addition, the FSQ-MIS also exhibited high parallel validity with DSM-5.

**Table 4:** Parameter estimation of General Loglinear Analysis.

Time	DSM-5 criterion				FSQ-MIS-F1			
	Estimation	Z	*P*	95% CI	Estimation	Z	*P*	95% CI
I	0.595	12.621	<0.001	0.502, 0.687	0.557	11.811	<0.001	0.464, 0.649
II	0.297	6.786	<0.001	0.211, 0.382	0.302	6.915	<0.001	0.217, 0.388
III	0.244	5.690	<0.001	0.160, 0.327	0.234	5.704	<0.001	0.160, 0.328
IV	0				0			

*Notes:* I, Dec 2019-May2020; II, Jun 2020-Nov 2020; III, Dec 2020-May 2021; IV, Jun 2021-Nov 2021.

## Discussion

In this study, we developed a new fast online screening questionnaire, FSQ-MIS, for identifying symptoms of different mental disorders during the COVID-19 pandemic. The FSQ-MIS exhibited high test-retest and split-half reliability, as well as satisfied construct, criterion-related, and discrimination validity. When applied to the COVID-19 dataset, the FSQ-MIS-F1 exhibited high parallel validity with DSM-5 in predicting the anxiety and depression trends during different pandemic stages.

The PCA analysis found 6 factors that could explain 54.28% variance. The depression and anxiety symptoms are mixed because the factor F1 consists of both depression and anxiety items. This coincided with the previous findings that the comorbidity of anxiety and depression is as high as 67% [17]. Hence, the FSO-MIS is not used to completely distinguish anxiety and depression. It is more appropriate to combine anxiety and depression into one factor F1 in this scale. The cognition factor (F2) is a general factor, which showed significant correlation with all other 6 criteria scales including HARS, HDRS, PSQI, PANSS, YBOCS, and BRMS. In clinical practice, the phenomenon of cognitive deficits is observed in almost all kinds of mental illness [18–22]. Even though the cognitive factor showed high power in identifying patients with normal controls (Fig. 1), the power in identifying disease categories was low. This was the reason why F2 was excluded from the ROC curves in factor prediction analysis (Fig. 2).

As for the performance in identifying mental disorders, the factors of FSQ-MIS show high discrimination and predictability. The factors derived from FSQ-MIS exhibit satisfying performance in identifying mental disorders from healthy controls (AUC from 0.73 to 0.98), indicating the high parallel validity with DSM-5. In addition, the factors could also identify specific mental disorder symptoms with high accuracy (AUC from 0.69 to 0.82).

In the present study, data collection spanned around two years after the COVID-19 outbreak. We divided the two years into four stages to investigate the trend of anxiety and depression proportion (including anxious and depressive status) during COVID- 19. The result presented two similar curves, which were discriminated by FSQ-MIS-F1 and DSM-5 respectively. The anxiety and depression proportions gradually declined during the two years, coinciding with previous work on regression of stress response [23]. Moreover, similar patterns of FSQ-MIS-F1 and DSM-5 prove the parallel validity of FSQ-MIS with DSM-5 criteria.

There were several limitations to this study that should be addressed: (1) Even though the FSQ-MIS is validated using a large-cohort dataset (*n* = 4812), all the participants were collected in one hospital in China, therefore, the efficiency of FSQ-MIS still needs to be further validated in multi-center studies; (2) The participants in this study are mainly young adults. More children and aged people should be included in the future study; (3) The questionnaire should be further tested in a larger sample of the general population to exam its sensitivity in screening mental disorder prevalence.

In summary, the present study developed a new fast online questionnaire that is efficient in screening mental illness symptoms during the COVID-19 pandemic. The reliability and validity test demonstrated its advantages in identifying and evaluating psychiatric symptoms. The combination of the proposed self-rating questionnaire with a smartphone application makes it easily accessible for users anytime. We believe the scale to be a useful tool for research and clinical practice.
